# Physical interaction and functional coupling between ACDP4 and the intracellular ion chaperone COX11, an implication of the role of ACDP4 in essential metal ion transport and homeostasis

**DOI:** 10.1186/1744-8069-1-15

**Published:** 2005-04-19

**Authors:** Dehuang Guo, Jennifer Ling, Mong-Heng Wang, Jin-Xiong She, Jianguo Gu, Cong-Yi Wang

**Affiliations:** 1 Center for Biotechnology and Genomic Medicine, Medical College of Georgia, 1120 15^th ^Street, CA4098, Augusta, GA 30912, USA; 2 Department of Oral and Maxillofacial Surgery, Mcknight Brain Institute and College of Dentistry, University of Florida, Gainesville, Florida, 32610, USA; 3 Department of Physiology, Medical College of Georgia, 1120 15^th ^Street, Augusta, GA 30912, USA

## Abstract

Divalent metal ions such as copper, manganese, and cobalt are essential for cell development, differentiation, function and survival. These essential metal ions are delivered into intracellular domains as cofactors for enzymes involved in neuropeptide and neurotransmitter synthesis, superoxide metabolism, and other biological functions in a target specific fashion. Altering the homeostasis of these essential metal ions is known to connect to a number of human diseases including Alzheimer disease, amyotrophic lateral sclerosis, and pain. It remains unclear how these essential metal ions are delivered to intracellular targets in mammalian cells. Here we report that rat spinal cord dorsal horn neurons express ACDP4, a member of Ancient Conserved Domain Protein family. By screening a pretransformed human fetal brain cDNA library in a yeast two-hybrid system, we have identified that ACDP4 specifically interacts with COX11, an intracellular metal ion chaperone. Ectopic expression of ACDP4 in HEK293 cells resulted in enhanced toxicity to metal ions including copper, manganese, and cobalt. The metal ion toxicity became more pronounced when ACDP4 and COX11 were co-expressed ectopically in HEK293 cells, suggesting a functional coupling between them. Our results indicate a role of ACDP4 in metal ion homeostasis and toxicity. This is the first report revealing a functional aspect of this ancient conserved domain protein family. We propose that ACDP is a family of transporter protein or chaperone proteins for delivering essential metal ions in different mammalian tissues. The expression of ACDP4 on spinal cord dorsal horn neurons may have implications in sensory neuron functions under physiological and pathological conditions.

## Background

Essential metal ions such as copper, manganese and cobalt are vital elements involved in functions of numerous enzymes and proteins in mammalian cells. Mammalian cells not only possess efficient uptake mechanisms to obtain these ions from their extracellular environment, but also have intracellular delivery system to translocate essential metal ions to specific enzymes and proteins. Deficiency in these essential metal ions affects normal cell functions, but they are toxic when present in excess. For example, they can damage DNA and proteins to induce cell death. Therefore, proper delivery of these essential metal ions into intracellular functional domains is vital. It is known that alteration of essential metal ion homeostasis is associated with diseases including Alzheimer's disease, amyotrophic lateral sclerosis, prion diseases, cataracts, mitochondrial disorders and Parkinson's disease [1-8]. Essential metal ion homeostasis also has important implications in sensory physiology, pathology and pain [9-11]. For example, copper is a cofactor for peptidylglycine a-amidating monooxygenase, an enzyme catalyzes the formation of a number of biologically active peptides including the pronociceptive peptide substance P [9]. Copper and manganese are cofactors for copper/zinc superoxide dismutase (Cu/ZnSOD) and Mn-superoxide dismutase (MnSOD), respectively [10,11]. These metal ion-dependent enzymes are involved in superoxide metabolism. Studies have shown that activity of these SOD enzymes can become abnormal during inflammation, which is an important underlying mechanism of pathological pain conditions and other neurological disorders [10,11].

Metal ion homeostasis is maintained through highly regulated processes, including transport, translocation, storage and secretion. Essential metal ions are transported into cells and then translocated to intracellular organelles to function as catalytic and structural cofactors for compartmentalized enzymes [12]. Although a number of ion transporters have been identified and characterized biochemically over the past several decades [13-18], the molecular identities of transporters for many metal ions are still elusive in mammalian cells. Unlike membrane ion channels permeable for ions such as Ca^2+ ^[19], essential metal ion transporters are coupled with intracellular metal ion chaperones [12,20-22]. Metal ion chaperones interact with transporters to receive metal ions, and then carry metal ions to target enzymes or proteins and donate metal ions to the targets. Several chaperones for copper ions have been identified in mammalian cells including COX17 and COX11 and they are shown to be essential for cell survival [20-22].

We have recently cloned and characterized a novel gene family named Ancient Conserved Domain Protein (ACDP) [23]. ACDP encodes four protein members (ACDP1-4) in both human and mouse [23,24]. The most prominent feature of ACDP gene family is the ancient conserved domain (ACD) found in evolutionarily divergent species ranging from bacteria, yeast, *C. elegans*, and *D. melanogaster *to mammals. ACDP proteins showed high amino acid homology to bacteria CorC protein (e.g., 35% AA identity with 55% homology), a protein believed to be involved in metal ion toxicity in bacteria [25]. Based on TopPred analysis it appears that ACDP can be a family of membrane proteins [24]. Consistent with this analysis, a recent study from our group has suggested that ACDP1, the first member of ACDP family, is localized close to or on the plasma membranes of hippocampus neurons [24]. Because ACDP4 was shown to have broader tissue distributions, including in both neuronal and non-neuronal tissues, we took ACDP4 as a start for exploring functions of ACDP family.

## Results

### Expression of ACDP4 proteins on spinal cord dorsal horn neurons

**Figure 1:**
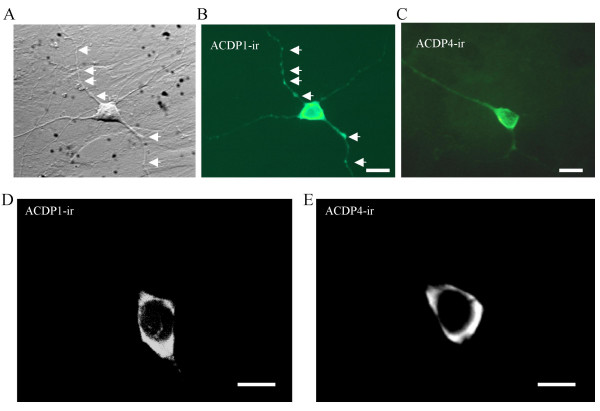
**Immunoreactivity of ACDP1 and ACDP4 on rat spinal cord dorsal horn neurons A. **The Micrograph shows a cultured dorsal horn neuron with its processes. The image was taken under a DIC (Nomarski differential interference contrast) microscope. **B. **ACDP1 immunoreactivity (ACDP1-ir) on the same neuron in A. Arrows indicate several punctuated sites of ACDP1-ir along dendrites. **C. **ACDP4-ir on another dorsal horn neuron. Scale bars: 10 μm. Two-week neuron cultures were used for the immunostaining. **D. **A confocal image of ACDP1-ir on a dorsal horn neuron. **E. **A confocal image of ACDP4-ir on anther dorsal horn neuron. The images were taken at the meddle sections of the cells. Scale bars were 20 μm for A, B and C and 10 μm for D and E.

We performed immunostaining of ACDP4 on the spinal cord dorsal horn neurons. ACDP1 immunostaining was also performed as additional information of ACDP family expression on dorsal horn neurons. Spinal cord dorsal horn neurons are CNS (central nervous system) neurons involved in somatosensory functions, including transmitting signals of nociceptive, mechanical, and thermal stimuli. Immunostaining was performed using polyclonal antibodies specific for ACDP1 and ACDP4, respectively. Both antibodies have been previously shown to have specific interactions with the corresponding target proteins [24]. We used cultured neurons grown on a monolayer of astracyte bedding for immunostaining. Therefore, cellular and subcellular distribution of ACDP1 and ACDP4 can be viewed clearly under a microscope. ACDP1 was expressed on both soma and dendrites of neurons (Fig. 1A &1B). Interestingly, ACDP1-ir on dendrites was shown to be punctuated (Fig. 1A &1B). ACDP4-ir was also found on the soma and dendrites of neurons, its distribution on dendrites was not punctuated (Fig. 1C). Neither ACDP1-ir nor ACDP4-ir was observed positively on astrocytes, the bedding underneath neurons. Confocal images showed that ACDP1-ir (Fig. 1D) and ACDP4-ir (Fig. 1E) were strongest at the edge of cells, suggesting that most ACDP1 and ACDP4 proteins were located close to and some of them maybe on the plasma membranes of spinal cord dorsal horn neurons.

### Interactions between ACDP4 and intracellular metal ion chaperone COX11

To explore the potential functions of ACDP proteins, we first searched whether any known proteins interact with them by screening a pretransformed human fetal brain cDNA library in a yeast two-hybrid system. This Matchmaker system is an advanced GAL4-based two-hybrid system that provides a transcriptional assay for detecting protein interactions *in vivo *in yeast. We used ACDP4 as a model in this study because it has broader tissue distribution than ACDP1. In the Matchmaker system ACDP4 (bait gene) was expressed as a fusion to the GAL4 DNA-binding domain (DNA-BD), which was used to screen a pretransformed human fetal Matchmaker cDNA library. When the bait and library fusion proteins interact, the DNA-BD and activation domain (AD) are brought into proximity, thus activating transcription of the reporter gene. In order to prevent non-specific interactions, we cultured the cells in the highest stringency condition. Among 2 × 10^6 ^transformants, only one clone was identified to strongly interact with ACDP4. The DNA for this clone was recovered from yeast and then transformed into XL-1 Blue competent cells (Stratagene). Individual clones were screened first by PCR and then sequenced using the primers from vector. We found that the yeast clone actually contains two plasmids, one plasmid is corresponding to the intracellular metal ion chaperone COX11, and the other one is an RNA binding motif protein 30 (RBM30) with unknown function.

**Figure 2:**
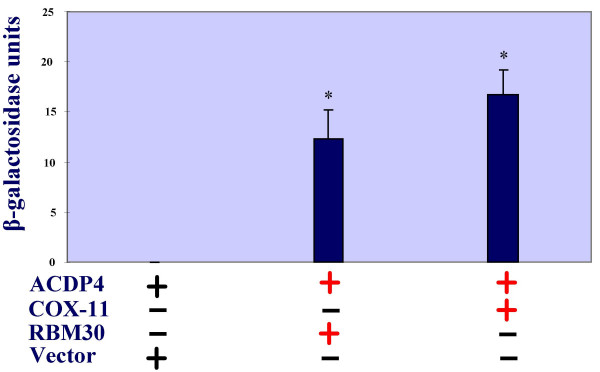
**Interactions of ACDP4 with COX11 or with RBM30 in a yeast two-hybrid system**. Cultures were assayed for β-galactosidase to verify two-hybrid interactions. β-galactosidase secreted from yeast containing the *lacZ *reporter gene after GAL4 activation were detected in the presence of chromogenic substrate ONPG (ortho-nitrophenyl β-D-galactopyranoside). Vector, empty pGADT7 vector.

To confirm the above results, we established full-length constructs for the pGADT7-COX11 and pGADT7-RBM30 (as targets). Subsequently, the pGBKT7-ACDP4 plasmid was co-transformed into an AH109 yeast strain either with the pGADT7-COX11 or the pGADT7-RBM30 plasmid. An empty pGADT7 vector was used as a control. The cultures were assayed for β-galactosidase to verify two-hybrid interactions (Fig. 2). In response to GAL4 activation, yeast containing the *lacZ *reporter gene secretes β-galactosidase, which can be detected in the presence of the chromogenic substrate ONPG (ortho-nitrophenyl β-D-galactopyranoside). When hydrolyzed by β-galactosidase, colorless ONPG turns to ONP (ortho-nitrophenyl), a yellow product. Therefore, enzyme activity of β-galactosidase can be measured by the rate of appearance of yellow color using a spectrophotometer. We found that ACDP4 strongly interacts with both COX11 and RBM30 (Fig. 2). On the other hand, there was no β-galactosidase activity detected in the controls, suggesting that the interactions detected are true. The interactions between ACDP4 and the metal ion chaperone COX11 provided an initial clue for a potential role of ACDP4 in metal ion homeostasis.

### The effects of ectopic expression of ACDP4 on metal ion toxicity in HEK293 cells

**Figure 3:**
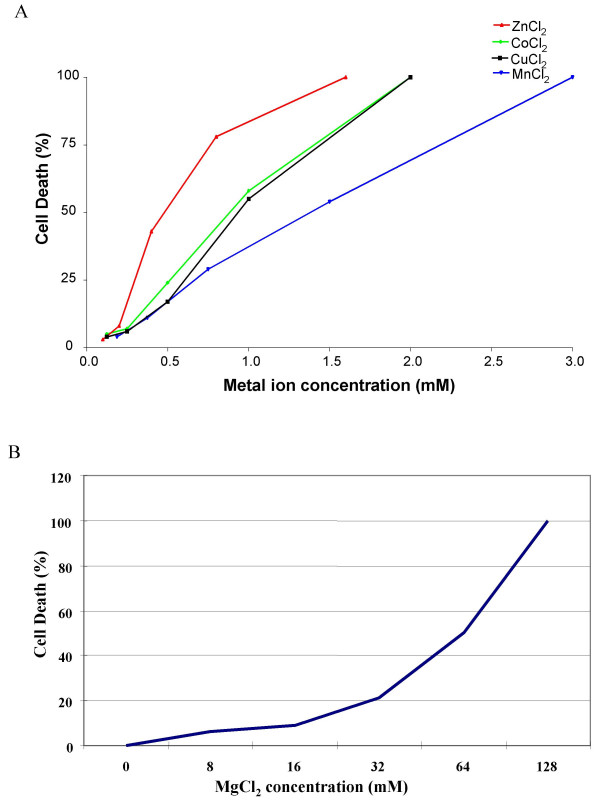
**Killing curve of HEK293 cells for selected divalent ions A. **Killing curves for Cu^2+^, Mn^2+^, Co^2+ ^and Zn^2+^. **B. **Killing curve for Mg^2+^. Cell death was estimated by counting a total of 300 cells in each field with 2–3 fields under microscope. Apparent EC_50 _values were 0.9 mM for Cu^2+^, 1.4 mM for Mn^2+^, 0.8 mM for Co^2+^, 0.6 mM for Zn^2+ ^and 64 mM for Mg^2+^.

Essential metal ions can produce oxidative toxicity when excessive amounts are accumulated inside cells. We first determined metal ion toxicity using HEK293 cells that were not transfected with ACDP4 plasmids. This serves as baseline of metal ion toxicity. Five divalent ions including Cu^2+^, Mn^2+^, Co^2+^, Mg^2+ ^and Zn^2+ ^were selected for the study. To establish a killing curve for each of the selected metal ions, HEK293 cells were plated at 3 × 10^5 ^per well in 6-well cultural plates and then cultured in medium containing different concentrations of CuCl_2_, MnCl_2_, CoCl_2_, MgCl_2 _and ZnCl_2 _for 48 hrs, respectively. The cells were then examined for viability by trypan blue staining. As shown in Fig. 3, the concentrations responsible for 50% of cell death (EC_50_) for CuCl_2_, MnCl_2_, CoCl_2 _and ZnCl_2 _were 0.9 mM, 1.4 mM, 0.8 mM and 0.6 mM, respectively (Fig. 3A). On the other hand, MgCl_2_, showed much less cell toxicity at low minimolar concentrations (Fig. 3B).

**Figure 4:**
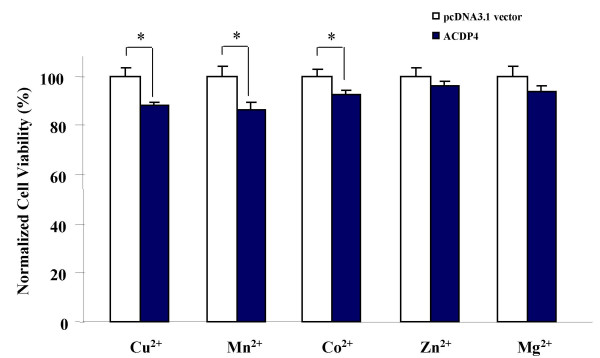
**The effects of ectopic expression of ACDP4 on metal ion toxicity in HEK293 cells**. ACDP4 is ectopically expressed in HEK293 cells using pcDNA3.1-ACDP4 plasmid transfection. Transfection of an empty vector (pcDNA3.1) was used as a control. Cells were cultured for 48 hrs in medium containing the 5 type of metal ions, and each type of metal ions was at its concentration of the apparent EC_50 _established in Figure 3. For normalization, the viability of control cells was scaled to 100%, and cell viabilities for cells transfected with ACDP4 were normalized in reference to the control cells. Data represent mean ± SEM, * P < 0.05, Student-*t *test.

Next, we tested whether ectopic ACDP4 expression may enhance metal ion toxicity in HEK293 cells. pcDNA3.1-ACDP4 plasmid was transfected into HEK293 cells using the Effectene Transfection Reagent. Transfection of an empty vector (pcDNA3.1) was used as a control. The cells were then cultured in medium containing the above metal ions each at its apparent EC_50 _identified above, and metal toxicity was measured at 48 hrs after the addition of metal ions. Ectopic ACDP4 expression significantly increased metal ion toxicity for cells cultured with Cu^2+^, Mn^2+ ^and Co^2+ ^(Fig. 4). The cell viabilities with ectopic ACDP4 in medium containing Cu^2+^, Mn^2+ ^and Co^2+ ^was 12% (*P *< 0.04), 14% (*P *< 0.02) and 8% (*P *= 0.05) lower than that of the cells transfected with empty vectors, respectively (Fig. 4). We did not observe any significant difference of cell viabilities between cells with ectopic ACDP4 and control cells when Mg^2+ ^or Zn^2+ ^were tested (Fig. 4). These results suggest that ACDP4 is probably preferential for Mn^2+^, Cu^2+ ^and Co^2+ ^over Mg^2+ ^and Zn^2+^.

### Enhanced metal ion toxicity by ectopic co-expression of ACDP4 and COX11

**Figure 5:**
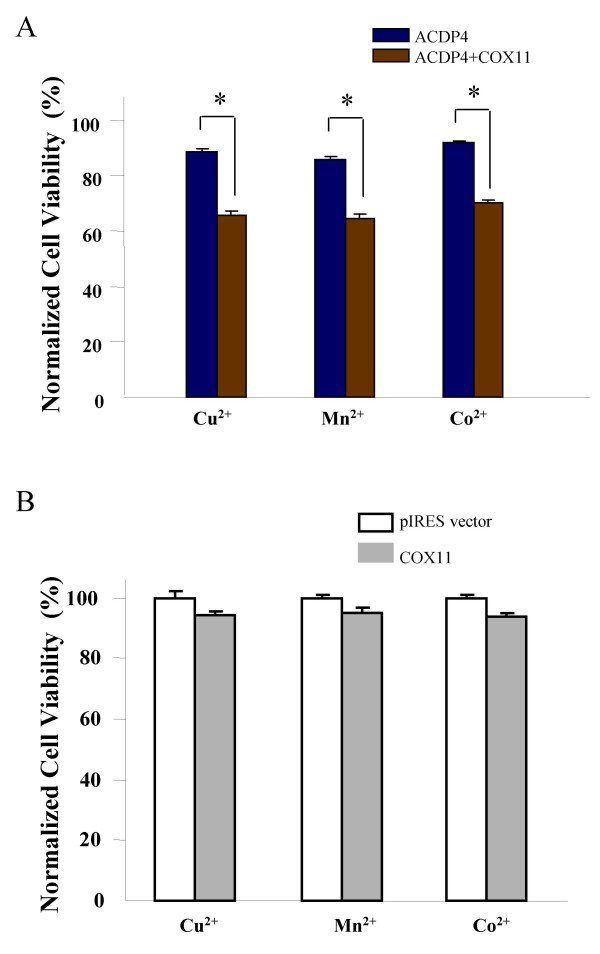
**Functional coupling of ACDP4 with COX11 in metal ion toxicity A. **The effects of ectopic co-expression of ACDP4 and COX11 on metal ion toxicity in HEK293 cells. Co-expression of ACDP4 and COX11 significantly enhanced metal ion toxicity. **B. **Metal ion toxicity for HEK293 cells ectopically expressed COX11 alone. There were no significant differences in cell viability between control cells and cells ectopically expressed COX11 alone.

While we showed physical interaction between ACDP4 and COX11 in a yeast two-hybrid system, it is more important to know whether ACDP4 may be functionally coupled with COX11 in mammalian cells. To address this question, we investigated metal ion toxicity in cells ectopically co-expressing ACDP4 and COX11. For this purpose, HEK293 cells were co-transfected with ACDP4-pcDNA3.1 plasmid along with COX11-pIRES-eGFP plasmid. The transfected cells were cultured in medium with the five metal ions at their apparent EC_50 _concentrations identified in Figure 3, and metal ion toxicity was examined after 48 hrs. Co-transfection of ACDP4 and COX11 significantly increased HEK293 cell toxicity to Cu^2+^, Mn^2+ ^and Co^2+ ^(Fig. 5A) but not to Zn^2+ ^and Mg^2+ ^(not shown). The cell viabilities in medium with Cu^2+^, Mn^2+ ^and Co^2+ ^were 32% (*P *< 0.006), 34% (*P *< 0.001) and 23% (*P *< 0.002) lower than the control cells, respectively (Fig. 5A). The cell death for Cu^2+^, Mn^2+ ^and Co^2+ ^were increased by 2.8 fold, 2.5 fold 2.9 fold, respectively, compared to transfection of ACDP4 alone (Fig. 4).

While tranfection of ACDP4 increased cell toxicity to Cu^2+^, Mn^2+ ^and Co^2+ ^(Fig. 4) and co-tranfection of ACDP4 with COX11 further enhanced cell toxicity to these ions (Figure 5A), transfection of COX11 alone had no significant effect on baseline metal ion toxicity (Figure 5B). These results together suggest a functional coupling between ACDP4 and COX11 for metal ion toxicity in HEK239 cells.

## Discussion

In the present study, we have demonstrated that ACDP4 physically interacts with COX11 and functionally coupled with this metal ion chaperone. We have shown that ectopic expression of ACDP4 enhances cell toxicity to several essential metal ions including Cu^2+^, Mn^2+ ^and Co^2+^, and that co-expression of ACDP4 with COX11 produces more pronounced increases of metal ion toxicity. This is the first study to explore potential functions of a member of ACDP protein family. The physical interaction and functional coupling with a metal ion chaperone indicates that ACDP4 is involved in the homeostasis and toxicity of essential metal ions.

The ACDP gene family is highly conserved in both human and mouse [23,24]. However, it has been complete unknown for their potential functions before the present study. Nevertheless, the sequence conservation and the presence of multiple members within a species imply a functional importance associated with this gene family. While we were completing this work, Yang *et al*. reported the involvement of a yeast ACDP homolog, Mam3p, in manganese homeostasis and toxicity in yeast [26]. Their results demonstrated that Mam3p operates independently to the well-established manganese trafficking pathways in yeast involving the manganese transporters, Pmrlp, Smf2p and pho84p [26]. In our present study, we have demonstrated the involvement of ACDP4 in copper and cobalt toxicity in addition to manganese. Furthermore, our functional results were obtained from mammalian cells.

**Figure 6:**
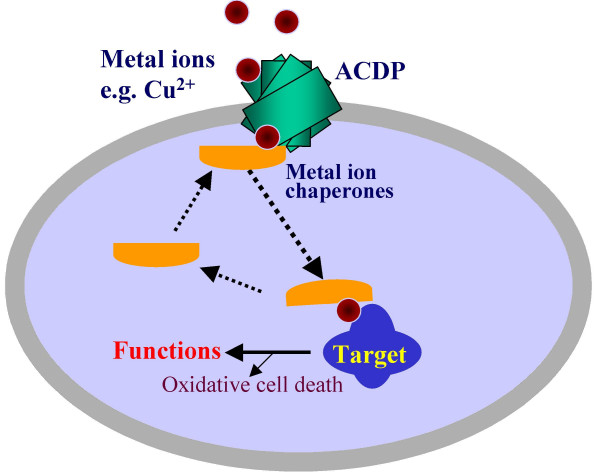
**A postulated model of ACDP-mediated metal ion delivery in mammalian cells**. An essential metal ion is first transferred through plasma membrane via an ACDP. An intracellular chaperone specifically interacts with the intracellular parts of ACDP. The chaperone receives the metal ion from ACDP and then carries the ion to a specific target protein. Overload of cells with essential metals results in oxidative cell death.

From our functional study using metal ion toxicity as a measure, it appears that ACDP4 functions at a site upstream to COX11. This idea is supported by three lines of evidence. First, ectopic expression of ACDP4 alone could enhance metal ion toxicity. Second, ectopic expression of COX11 alone did not affect metal ion toxicity. Third, co-expression of ACDP4 with COX11 produced more pronounced metal ion toxicity than ectopic expression of ACDP4 alone. Interactions between ACDP4 and COX11 suggest that ACDP4 may be a metal ion transporter in mammalian cells (Fig. 6). This is because a metal ion chaperone normally interacts with upstream metal ion transporters to receive metal ions and interacts with downstream target proteins to deliver metal ions. However, a direct study on ACDP4-mediated metal ion accumulation in cells is needed to confirm transport functions of ACDP4. Alternatively, ACDP4 may be an intermediate metal ion chaperone upstream to COX11. Detailed studies in the future on ACDP4 membrane localization and its interactions with metal ion chaperones in mammalian cells will provide more insights into the mechanisms by which ACDP4 is involved in metal ion homeostasis and toxicity. It would also be interesting to study whether other members of ACDP family may be involved in essential metal ion homeostasis and toxicity in mammalian cells. A particular interesting issue is the potential neuronal functions of ACDP1 since it is almost exclusively expressed in CNS neurons in the brain (24) and the spinal cord neurons (Fig. 1).

Interactions between ACDP4 and COX11 provide a structural and functional linkage to COX11. COX11 is an intracellular copper chaperone originally identified in yeast as essential for cytochrome *c *oxidase activity and heme *α *stability in subunit I. [20]. A recent study suggested a role for this protein in formation of the binuclear copper-heme center [21]. Spectroscopic and mutagenesis studies indicate that the copper ion in COX11 is ligated by three conserved cysteine residues [22]. The C-terminal domain of this protein forms a dimer that coordinates a single CuC per monomer. While COX11 was found to be a dimer, it remains possible that copper transfer to and from COX11 occurs through heterodimeric interaction with other proteins [22]. If ACDP4 is confirmed to be a metal ion transporter or an upstream metal ion chaperone, it would be interesting to know how ACDP4 delivers metal ions to COX11, whether ACDP4 and COX11 are co-localized on or near the plasma membranes, whether ACDP4 and COX11 are also co-localized on the membranes of other intracellular organelles such as mitochondria. We have shown that ACDP4 also interacts with RBM30, an RNA binding motif protein 30. RBM30 contains a zinc knuckle, raising a possibility that it may serve as an intracellular zinc chaperone. However, we did not observe an enhanced toxicity to zinc in cells transfected with RBM30 or co-transfected with both ACDP4 and RBM30. Therefore, it remains to determine whether interaction of ACDP4 with RBM30 may have any biological significance.

The expression of ACDP4 and ACDP1 on dorsal horn neurons of the spinal cord shown in the present study may have implications in sensory physiology and pathology. Both ACDP4-ir and ACDP1-ir were found to be localized close to the plasma membranes of dorsal horn neurons. If ACDP4 and ACDP1 are metal ion transporters or chaperones in these neurons, they should be involved in regulating homeostasis of essential metal ions in the spinal cord dorsal horn neurons. Essential metal ions are essential for activity of many enzymes, including those for synthesis of neuropeptides (e.g. substance P) and neurotransmitters (e.g. monoamine) in the spinal cord [9], and those for superoxide metabolism in peripheral sensory nerves and CNS sensory neurons in the spinal cord [10,11]. Abnormal superoxide metabolism has been shown to be a critical factor in inflammation and pathological pain conditions [10,11]. It is conceivable that through regulating essential metal ion homeostasis, ACDP4 and perhaps also other ACDP members can affect sensory process including nociception. It is also predictable that the functions of ACDP family will go beyond somatosensory system.

## Methods

### Neuronal cell preparation and immuostaining

Sprague-Dawley rats were used according to the Institutional Animal Care and Use Committee guideline of the University of Florida. Dorsal horn neuron cultures were prepared as described previously. [27]. In brief, spinal cord dorsal horns were dissected out from rat embryos at the age of 16 days *in utero *(E16). Dorsal horns were incubated separately for 25 min at 37°C in S-MEM medium (Gibco, Grand Island, NY) with 2.5% trypsin (Gibco) and then triturated to dissociate neurons. The neurons were plated on glass coverslips previously prepared with a monolayer of rat cortical astrocytes. Neurons were maintained in MEM (Gibco) culture medium that contained 5% heat-inactivated horse serum (JRH Biosciences, Lenexa, KS), uridine/5-flouro-2'-deoxyuridine (10 μM; Sigma, St. Louis, MO), 8 mg/ml glucose and 1% vitamin solution (Gibco). The cultures were maintained at 37°C in a humidified atmosphere of 95% air and 5% CO_2_, and were fed weekly with fresh culture medium.

Neurons were used for immunostaining of ACDP1 and ACDP4 at two weeks in culture. For immunostaining, neuronal cells on the coverslips were first fixed in PBS containing 4% paraformaldehyde (PFA) for 12 hrs at 4°C and then incubated in a solution containing 4% PFA and 0.4% Triton X-100 at 4°C for 1 hr. After washing with PBS three times, the cells were incubated with a blocking solution containing 1:30 normal goat serum, and subsequently incubated with a rabbit polyclonal anti-ACDP antibody (1:3000) overnight at 4°C. After extensive washing with 1% goat serum PBS solution, the cells were incubated with an Alex 488 conjugated secondary antibody (1:100 in 1% goat serum PBS solution, Molecular Probes) for 3 hrs at room temperature. Following final washes with 1% goat serum PBS solution, the neuronal cells on the coverslips were cover-slipped with a glycerol-based anti-photobleach medium. The cells were viewed under a fluorescence microscope (Olympus) with a 40X oil-immersion objective or a confocal fluorescence microscope (Carl Zeiss) with a 60X objective.

### Establishment of killing curve

HEK293 cells were plated at 3 × 10^5 ^per well of 6 well plates. The cells were cultured for 48 hrs in medium containing different concentrations of MgCl_2_, CuCl_2_, ZnCl_2_, MnCl_2 _and ECoCl_2_, respectively. Dead cells were then stained with trypan blue solution at a ratio of 4:6 (Gibco). The percentage of cell death was determined by counting a total of 300 cells in each field with 2–3 fields under microscope.

### Plasmid construction

Full-length ACDP4 gene was cloned into the pGBKT7 and pcDNA3.1 vectors using the *EcoR I *cutting site. COX11 and RBM30 were amplified from fetal brain cDNA and then cloned into pIRES-eGFP vector with *EcoR I *and *BamH I*, and *Xho I *and *BamH I *cutting sites, respectively.

### Yeast two-hybrid analysis

The Matchmaker Galt4 two-hybrid system 3 kit (Clontech) was used for two-hybrid analyses. The ACDP4 coding sequence was PCR engineered and cloned into the pGBKT7 vector, which was used as a bait to screen a pretransformed human fetal brain Matchmaker cDNA library at high stringency culture condition. To confirm the interaction between ACDP4 and COX11 or RBM30, the full-length COX11 and RBM30 cDNA were cloned into the pGADT7 vector (as targets), which was then co-transfected into an AH109 yeast strain along with the pGBKT7-ACDP4 plasmid, respectively. An empty pGADT7 vector was used as a control. The cultures were assayed for β-galactosidase to verify two-hybrid interaction according to the manufacturer's instruction.

### Cell culture and transfection

HEK293 cells were cultured in Dulbecco's modified Eagles's medium (Mediatech, Inc.) supplemented with 10% fetal bovine serum and 100 units/ml antibiotic-antimycotic (Invitrogen). Transfections were carried out using 2 ug of plasmid DNA/100-mm dish and Effectene Transfection Reagent according to the instructions of the manufacturer (Qiagen).

### Metal toxicity assay

We used an in vitro toxicology assay kit (Sigma) for measurement of metal toxicity according to the manufacturer's instruction. Cell viability estimated by this *in vitro *toxicology assay determines cell number spectrophotometrically as a function of mitochondrial activity in living cells. Briefly, the cells were cultured with indicated metal ion for 44 hrs and then supplemented with 20 μl MTT per well. The cells were cultured for additional 4 hrs. Subsequently, the cultural plates were spun for 5 min at 1000 rpm to remove the supernatant. The plates were dried in an incubator for about 4 hrs. 200 μl of MTT solubilization solution was then used to dissolve the resulting formazan crystals. The results were read at 570 nm after 6 hrs incubation with a Synergy HT plate reader (Bio-Tek).

### Data analysis and statistics

Unless otherwise indicated, data represent mean ± SEM, p < 0.05, student-*t *test.

## Competing interests

The author(s) declare that they have no competing interests.
